# Major discrepancy between clinical and autopsy diagnosis: a descriptive single-center analysis and its implications for the South African public health system

**DOI:** 10.3389/fmed.2026.1884301

**Published:** 2026-07-23

**Authors:** Moshawa C Khaba, Khomotso Maaga, Ntebogeng Kgokong

**Affiliations:** 1 Department of Anatomical Pathology, Dr George Mukhari Tertiary Laboratory, National Health Laboratory Service, Sefako Makgatho Health Sciences University, Pretoria, South Africa; 2 Department of Public Health, Sefako Makgatho Health Sciences University, Pretoria, South Africa

**Keywords:** autopsy, major discrepancy, Goldman criteria, clinical audit, public health

## Abstract

**Background:**

A global decline in the number of clinical post-mortems sought and conducted indicates a diminishing necessity for post-mortem exams. Notwithstanding advancements in medical technology, autopsies continue to serve as a pertinent method for ascertaining the cause of death.

**Objectives:**

This study aimed to identify prevalent major misdiagnoses in clinical settings through autopsy and to investigate potential contributing factors.

**Methods:**

A retrospective cross-sectional study was conducted, including 51 autopsies retrieved from the NHLS Laboratory Information System (LIS) and classified as either Class I or Class II discrepancies according to the Goldman criteria. Stata-18 was used to analyse descriptive data and examine the associations between clinicopathological factors and the major discrepancy classes.

**Results:**

Of the 51 autopsies included in the study, 68.63% (*n* = 35) were female in contrast to 31.37% (*n* = 16) of males, with a mean death age of 39.3 years. A total of 88.24% (*n* = 45) of the cases were classified as Class I discrepancies, and 11.76% (*n* = 6) were Class II. There was no evidence of an association between discrepancies and age, sex and disease type. However, autopsy diagnosis showed a statistically significant association with Class I discrepancies (*p* ≤ 0.05) accounted for the largest proportion of misdiagnoses, with pulmonary disease being the most frequently misdiagnosed condition at 44.44% (*n* = 20).

**Conclusions:**

Autopsies remain a pertinent clinical audit tool despite advances in medical technology. The hospital should provide funds for autopsies and encourage doctors to utilize them as a clinical audit instrument to enhance patient management.

## 1 Introduction

Autopsies are still considered the gold standard for determining the accuracy of one's cause of death. Although autopsies were previously regarded as essential and routinely used, their prevalence has been declining globally (1–3). This decrease has been attributed to a variety of factors, including advances in disease diagnosis, which have led to greater reliance on medical technology. Additionally, family refusal for consent to conduct an autopsy due to traditional and religious beliefs, and the reluctance of anatomical pathologists to perform the autopsy for a variety of reasons, are other factors associated with this decline (4, 5). It has been observed that the decline in autopsies coincides with an increase in major clinical diagnostic errors, a public health concern (1, 2, 4).

Despite significant advancements in diagnostic medicine, discrepancies between clinical and autopsy findings remain common. Autopsies continue to serve an important role in validating clinical diagnoses and providing an objective measure of diagnostic accuracy in evidence-based medicine (4, 5). They remain the most reliable method for confirming clinical diagnoses and identifying missed or incorrect diagnoses that may have clinical significance.

Diagnostic discrepancies are commonly classified using the Goldman criteria into major and minor categories. Major discrepancies (Classes I and II) represent clinically significant diagnostic errors. Class I discrepancies refer to missed diagnoses that, if identified earlier, could have altered therapy and potentially improved survival. Class II discrepancies involve missed diagnoses that would not have changed patient outcome. Minor discrepancies (Classes III–V) include findings that may or may not have contributed to death, as well as cases with complete diagnostic agreement (6, 7).

Reported major discrepancy rates in the literature vary widely, ranging from approximately 14 to 58%, highlighting ongoing concerns regarding diagnostic accuracy in clinical practice (8, 9). These discrepancies may arise from disease complexity, variability in clinician experience, resource limitations, and inconsistent consideration of autopsy as a diagnostic and quality assurance tool (7, 10). While not all discrepancies represent diagnostic error, many reflect clinically important gaps in recognition of disease processes and comorbid conditions (10–12).

Consequently, there is a need to generate greater awareness and sustained academic interest in the clinical significance of diagnostic discrepancies, thereby encouraging further autopsy-based research in this field. Despite extensive global literature on clinical–autopsy diagnostic discrepancies, limited data exist describing the magnitude and nature of these discrepancies within our local setting (9, 10, 13). This study therefore aims to address this local knowledge gap by determining the diagnostic discrepancy rate and characterizing the nature of missed diagnoses in a quaternary-level hospital while situating the findings within the broader regional and global context to facilitate meaningful comparison with existing literature.

## 2 Methods and materials

The study utilized a retrospective descriptive study design comprising of autopsies performed at our center between 01 January 2013 and 31 December 2018. This study period was selected to represent a stable pre-pandemic baseline, ensuring consistency in clinical practice, diagnostic workflows, and autopsy activity. The COVID-19 pandemic period was deliberately excluded to avoid potential confounding effects, as significant disruptions in healthcare delivery, case selection, and autopsy services occurred during this time, which may have influenced clinicopathological correlation outcomes.

Only cases with complete medical records consisting of clearly defined antemortem and postmortem diagnoses were included in the study. Cases with insufficient clinical data, uncertain autopsy diagnosis, and medico-legal/forensic postmortems were excluded.

TrackCare, a laboratory information system (LIS) from the National Health Laboratory Service (NHLS), was used to retrieve clinicopathological data, including demographics (gender, age), the requesting department, and ante-mortem and post-mortem diagnoses.

Comparisons between the ante-mortem and post-mortem diagnoses were assessed using the Goldman classification (8) (Table 1). This tool has been used to detect discrepancies between ante-mortem and post-mortem diagnoses and has been applied both globally and locally, thereby affirming its validity and reliability. For this study, only major discrepancy categories (Class I, Class II) were assessed and included.

**Table 1:** Goldman classification of diagnostic discrepancies.

Class	Definition
Class I	Missed major diagnosis that, if detected before death, would likely have changed management and resulted in prolonged survival or cure.
Class II	Missed major diagnosis that, if detected before death, would not have changed management or survival.
Class III	Missed minor diagnosis related to the terminal disease but not directly contributing to death.
Class IV	Missed minor diagnosis unrelated to the cause of death.
Class V	No discrepancy between clinical and autopsy diagnoses (complete agreement).

In cases where no formal microscopic report was available on the system, haematoxylin and eosin (H&E)-stained slides were retrieved from the NHLS archives for appraisal to reach a final autopsy diagnosis. Moreover, when H&E-stained slides were missing from storage, formalin-fixed, paraffin-embedded (FFPE) tissue blocks were retrieved, cut according to departmental protocols, and the slides were reviewed independently by the principal investigator.

## 3 Data analysis

The data were collected, cleaned, and coded in Microsoft Excel, then exported to STATA-18, a statistical software package developed by StataCorp in College Station, TX, USA, for analysis. Descriptive data were presented using frequencies and percentages for categorical variables, and means with corresponding standard deviations for continuous variables. Fisher's exact test was used to examine associations between clinical variables and the Goldman classification, with a significance threshold of *p* ≤ 0.05. Continuous variables such as age were categorized into two groups ( ≤ 18 years and >18 years) to satisfy the statistical requirements of the applied tests (Fisher's exact test and chi-square tests of association) and to facilitate categorical analysis of associations between clinical variables and discrepancy class.

## 4 Results

A total of 267 autopsies performed during the study period were initially identified. After applying the inclusion criteria (*n* = 154), cases that did not meet the complete clinical and autopsy data requirements were excluded, yielding a final analytic sample of 51 cases.

### 4.1 Patient profile

#### 4.1.1 Sex of participants

**Figure 1:**
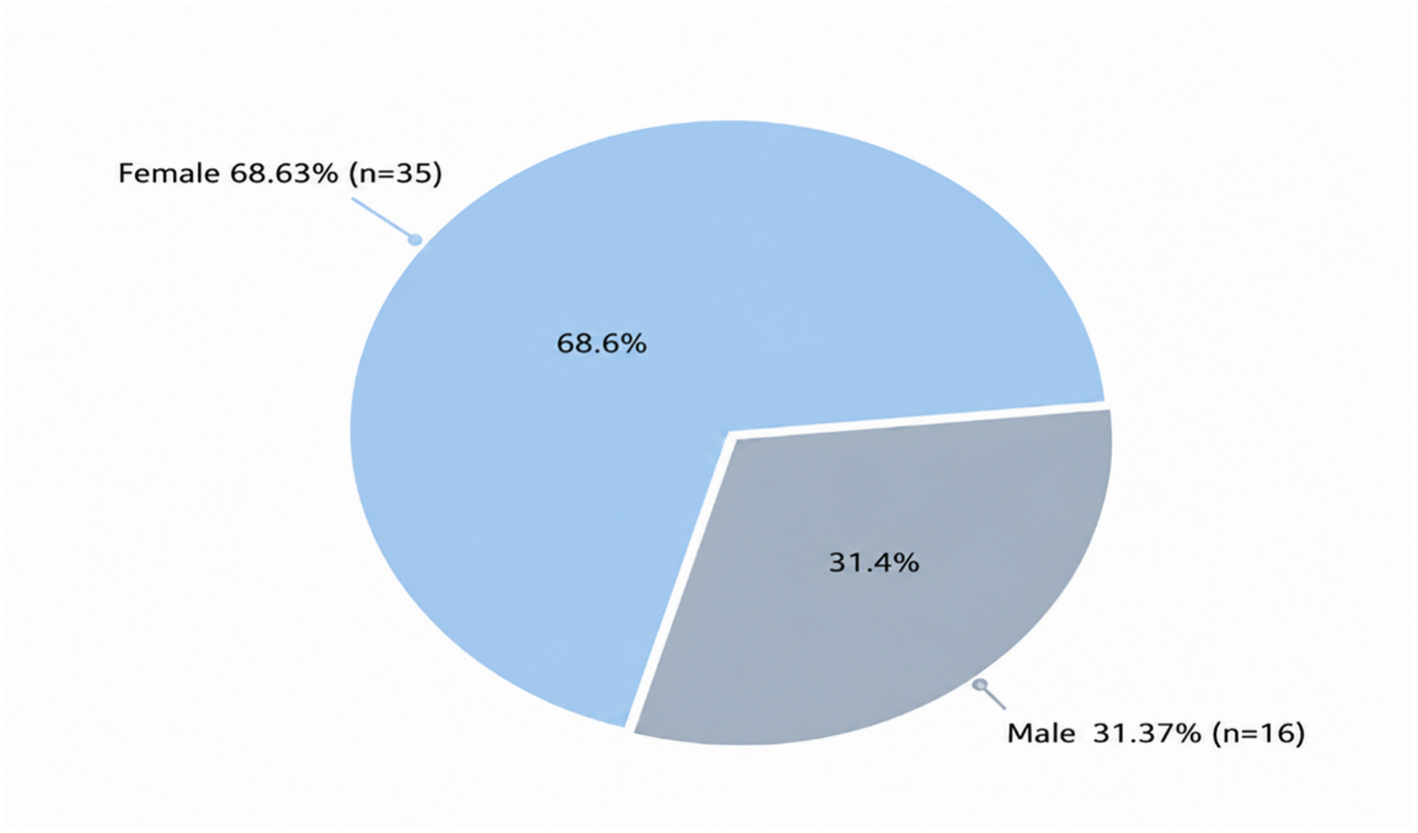
Sex distribution of cases.

The study included 51 patients, with a male-to-female ratio of 1:3. Females constituted the majority of the sample at 68.63% (*n* = 35), compared to males at 31.37% (*n* = 16) (Figure 1).

#### 4.1.2 Age of participants

**Figure 2:**
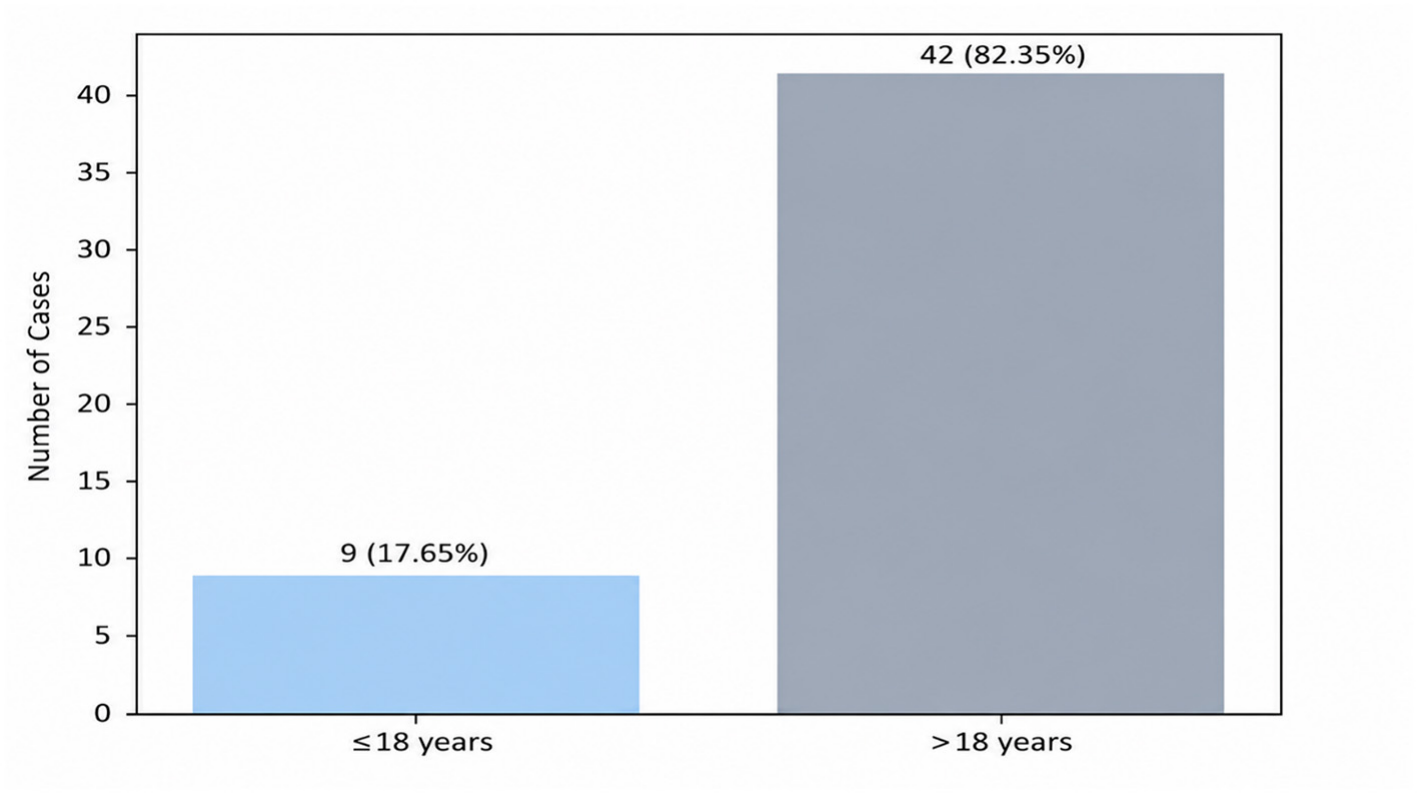
Age distribution of cases.

The mean age at death was 39.2 years (SD = 22.2), and the majority of the sample were older than 18 years (Figure 2).

### 4.2 Requesting department

**Figure 3:**
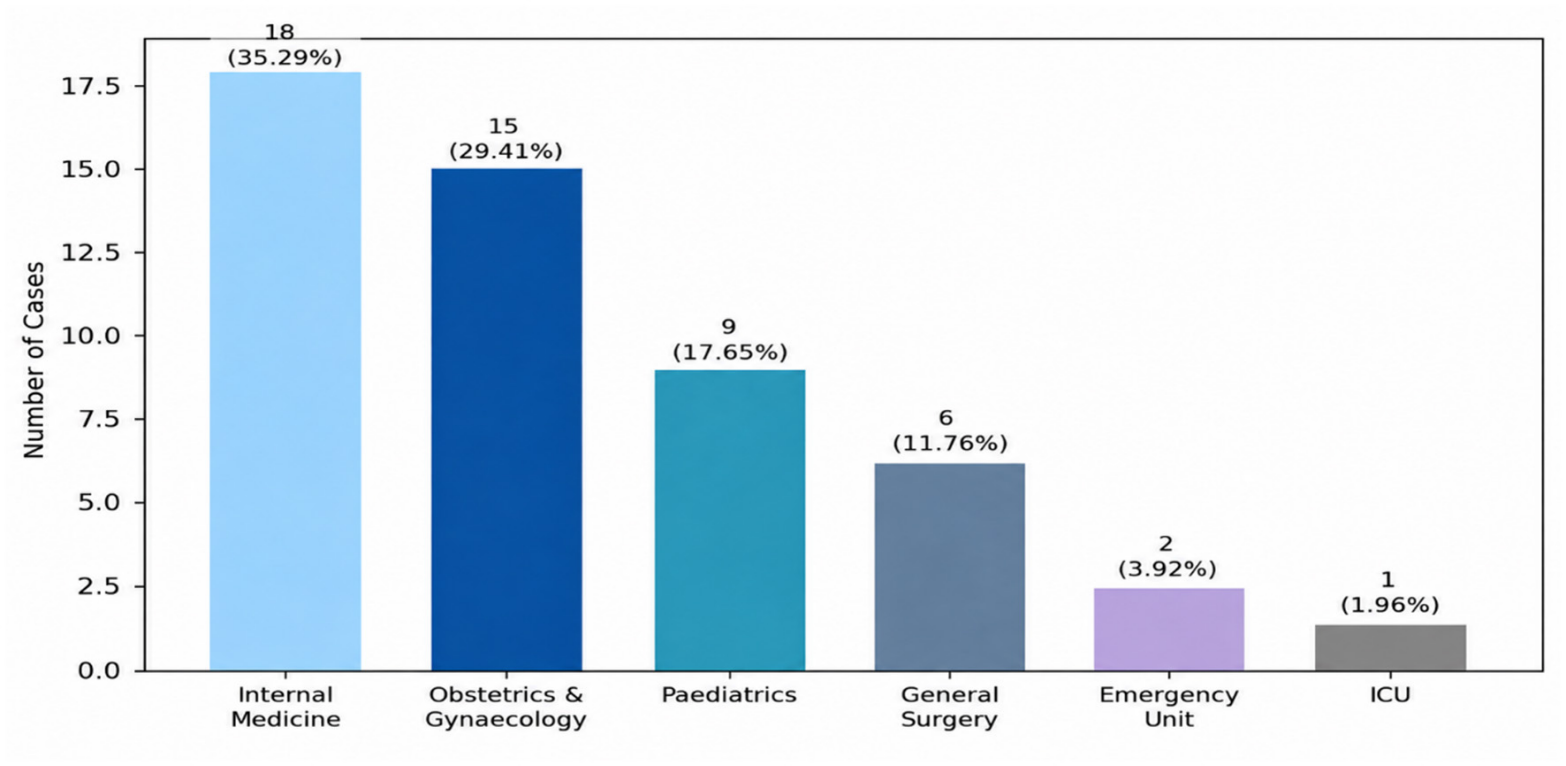
Department requesting autopsy.

The majority of autopsy requests originated from the departments of internal medicine (35.29%, *n* = 18), obstetrics and gynecology (29.41%, *n* = 15), and pediatrics (17.65%, *n* = 9). Fewer requests were received from general surgery (11.76%, *n* = 6), the emergency unit (3.92%, *n* = 2), and the intensive care unit (1.96%, *n* = 1), as illustrated in Figure 3.

### 4.3 Major discrepancy class

Class I discrepancies were more frequent at 88.24% (*n* = 45) compared to Class II discrepancies at 11.76% (*n* = 6). More information is provided in Table 2.

**Table 2:** ante-mortem and post-mortem diagnoses among cases with major diagnostic discrepancies.

Case	Ante-mortem (clinical) diagnosis	Post-mortem (autopsy) diagnosis	Goldman criteria (class)
1	Mixed mitral valve disease	Pulmonary embolus	I
2	Myocardial infarction	Congestive Heart Failure	I
3	Renal failure	Atrial myxoma	I
4	Nephropathy with refractory anemia	Interstitial lung disease	I
5	Disseminated Kaposi's sarcoma with DIC	Plasmablastic lymphoma	I
6	Abdominal sepsis	Chronic renal failure	I
7	Pulmonary embolus	Arrhythmogenic right ventricular cardiomyopathy	I
8	Disseminated tuberculosis	Endometrial adenocarcinoma	I
9	Gastroenteritis	Thromboembolic disease	I
10	Pulmonary embolus	Ruptured uterus	I
11	Ruptured ectopic pregnancy	Choriocarcinoma	I
12	Pulmonary embolus or myocardial infarct	Hypertrophic cardiomyopathy	I
13	Sudden death	Pulmonary embolus	I
14	Pulmonary embolus	Endometrial Endometrioid adenocarcinoma	I
15	Severe hypoglycaemia	Malignant hypertensive disease	I
16	Pneumonia	Thromboembolic disease	I
17	Pulmonary tuberculosis	Metastatic Basal Cell Carcinoma	I
18	Cerebral hemorrhages	Malignant hypertensive disease	I
19	Obstructive jaundice	Mucinous adenocarcinoma of the pancreas	II
20	Pulmonary tuberculosis	Plasmablastic lymphoma	II
21	Liver malignancy	Adenocarcinoma of head of pancreas tumor	II
22	Hypovolemic shock	Poorly differentiated carcinoma	II
23	Pancytopenia	Advanced Langerhan Cell Histiocytosis	II
24	NSAID's gastritis	Thromboembolic disease	I
25	Dysmorphic	Broncopneumonia	I
26	Aplastic anemia	Primary immunodeficiency syndrome with secondary infection	I
27	Liver failure secondary to biliary atresia	Tertiary syphilis with liver cirrhosis	I
28	Stroke	Multilobar pneumonia	I
29	Acute renal failure	Bronchopneumonia	I
30	Diabetic ketoacidosis	Bacterial meningitis	I
31	Nephroblastoma	Bronchopneumonia	I
32	Acute abdomen	Small bowel perforation	I
33	TB and sepsis	Multilobar pneumonia	I
34	Renal failure	Bronchopneumonia	I
35	Acute renal failure in pregnancy	Bronchopneumonia	I
36	Neonatal jaundice, apnoeic attacks, metabolic acidosis	Bronchopneumonia	I
37	Respiratory distress	Disseminated cryptococcosis	I
38	SLE and pancytopenia	Bronchopneumonia	I
39	Myocardial infarction	Multilobar pneumonia	I
40	Renal failure	Infective endocarditis	I
41	Complex ovarian mass	Perforated strangulated hernia	I
42	Deep venous thrombosis	Incarcerated hernia	I
43	Renal failure	Disseminated Mucormycosis	I
44	Pulmonary embolus	Ruptured uterus	I
45	Liver and renal impairment	Multilobar pneumonia	I
46	Atonic uterus and postpart haemorrhage with hypovolemic shock	Bronchopneumonia	I
47	Complicated TB	Disseminated cryptococcosis	I
48	PE	Multilobar pneumonia	I
49	Myocardial infarction or PE	Multilobar pneumonia	I
50	Respiratory failure secondary to infection	Perforated necrotic bowel tumor	I
51	Pulmonary embolus	Adenocarcinoma of the Pancreas	II

### 4.4 Disease classification

The three most common disease classifications according to the International Classification of Diseases (ICD) were pulmonary diseases (39.22%, *n* = 20), malignant diseases (19.61%, *n* = 10), and cardiovascular diseases (13.73%, *n* = 7). The remaining disease classifications included infectious diseases (9.80%, *n* = 5), gastrointestinal diseases (7.84%, *n* = 4), other conditions (5.88%, *n* = 3), and gynecological diseases (3.92%, *n* = 2). Further details are presented in Table 3.

**Table 3:** Disease classification as per International Classification of Diseases (ICD).

Disease (ICD)	Autopsy diagnosis	Frequency	Percentage
Pulmonary disease	Bronchopneumonia	20	39.22
	Interstitial lung disease		
	Multilobar pneumonia		
	Pulmonary embolus		
	Thromboembolic disease		
Cardiovascular disease	Arrhythmogenic right ventricular cardiomyopathy	7	13.73
	Congestive heart failure		
	Malignant hypertensive disease		
	Hypertrophic cardiomyopathy		
	Infective endocarditis		
Malignant tumor disease	Adenocarcinoma pancreas	10	19.61
	Adenocarcinoma endometrium		
	Choriocarcinoma		
	Poorly differentiated carcinoma		
	Plasmablastic lymphoma		
	Metastatic basal cell carcinoma		
Gastrointestinal disease	Small bowel perforation	4	7.84
	Perforated necrotic bowel		
	Incarcerated hernia		
	Perforated strangulated hernia		
Gynecological disease	Ruptured uterus	2	3.92
Infectious disease	Bacterial meningitis	5	9.80
	Syphilis		
	Disseminated cryptococcosis		
	Disseminated mucormycosis		
Others	Chronic renal failure	3	5.88
	Atrial myxoma		
	Advanced Langerhans cell histiocytosis		

Fisher's exact test showed no evidence of an association between discrepancies and age, sex, disease type, or clinical diagnosis. However, autopsy diagnosis showed a statistically significant association with discrepancies (*p* = 0.002), with pulmonary diseases (*n* = 20, 44.44%) more frequently misdiagnosed under Class I and malignant diseases equally misdiagnosed in both classes. Table 4 bears further reference.

**Table 4:** Factors associated with major discrepancies.

Factor	Frequency (%)	Discrepancy class (%)		*p*-value
		Class I	Class II	
Age at death (Mean 39.2; SD 22.2; Min 0; Max 85)				0.716
≤ 18	9 (17.65)	8 (15.69)	1 (1.96)	
>18	42 (82.35)	37 (72.54)	5 (9.80)	
Gender				0.424
Male	16 (31.37)	13 (25.49)	3 (5.88)	
Female	35 (68.63)	32 (62.75)	3 (5.88)	
Autopsy diagnosis as per ICD classification				0.002^*^
Cardiovascular Diseases	7 (13.73)	7 (15.56)	0	
Gastrointestinal diseases	4 (7.84)	4 (8.89)	0	
Infectious diseases	5 (9.08)	5 (11.11)	0	
Malignant diseases	10 (19.61)	5 (11.11)	5 (83.33)	
Gynaecological disease	2 (3.92)	2 (4.44)	0 (0.0)	
Pulmonary diseases	20 (39.22)	20 (44.44)	0	
Others	3 (5.88)	2 (4.44)	1 (16.67)	
Disease type				0.566
Communicable disease	28(54.90)	25(49.02)	3(5.88)	
Non-communicable disease	23(45.10)	20(39.22)	3(5.88)	

Fisher's exact test; *p* ≤ 0.05; 95 % confidence interval. ^*^Indicates statistical significance.

## 5 Discussion

This study examined 51 autopsy cases selected from an initial 267 records and identified a major clinical–autopsy discrepancy rate of 19.1%. Class I discrepancies were predominant (88.24%), indicating that in most cases the correct diagnosis, if made earlier, would have altered clinical management and potentially improved survival. Pulmonary diseases were the most common cause of death (39.22%) and were significantly associated with Class I discrepancies (*p* = 0.002), while malignant and cardiovascular conditions also contributed substantially. No significant associations were found between discrepancy class and age, sex, disease type, or clinical diagnosis, although autopsy diagnosis showed a statistically significant association with discrepancy classification.

The findings of this study are particularly important in the context of the persistently low rate of autopsy performance in Africa, including South Africa, and the concerning scarcity of research investigating trends and discrepancies between clinical and post-mortem diagnoses. The autopsy rate at a healthcare facility directly reflects its dedication to quality control and diagnostic precision, as the post-mortem review is the ultimate assessment of clinical care. Autopsies provide essential feedback by validating the cause of death and uncovering diagnostic errors in patient care; this process is essential for learning and improvement on a large scale (6).

In addition, autopsies are considered a key aspect of public health, as they improve health systems by providing accurate mortality statistics that support policymaking and by revealing important information about new and natural disease courses, which directly supports research and development (9).

This study revealed a major discrepancy rate of 19.1% between clinical and autopsy diagnoses, calculated as the proportion of major discrepancy cases (*n* = 51) among the total number of autopsies performed during the study period (*n* = 267). This finding is consistent with both local and international studies investigating discrepancies between clinical and autopsy diagnoses. Notably, the major discrepancy rate observed in the current study closely mirrors the pooled global estimate of 19.3% reported in a recent systematic review and meta-analysis of intensive care unit autopsy studies. The persistence of a major discrepancy rate of approximately 1 in 5 cases remains concerning and highlights the ongoing challenges in achieving diagnostic accuracy, despite advances in modern imaging, laboratory investigations, and other diagnostic technologies. These findings underscore the continued value of autopsy examination as a quality assurance tool for evaluating clinical diagnostic performance.

Furthermore, the overwhelming predominance of Class I discrepancies (88.24%) over Class II discrepancies (11.76%) indicates that the majority of discrepant diagnoses were such that earlier recognition and appropriate treatment might have altered patient management and potentially improved survival. This observation is particularly significant because Class I discrepancies constitute the most clinically important category of diagnostic error. These findings align with the existing literature, which demonstrates considerable geographic variation in major discrepancy rates. A global study involving 42 countries reported an overall major discrepancy rate of 19.3%, with rates ranging from 15 to 35% in European countries such as Germany, Spain, France, and the Netherlands. In contrast, higher rates of 35–45% have been reported in sub-Saharan Africa (Uganda and Ethiopia) and Southern Africa (South Africa). Across various geographical settings, a substantial proportion of major discrepancies involve potentially treatable conditions that were not recognized during life, underscoring the continued value of post-mortem examinations in evaluating diagnostic accuracy (9, 13–19).

A recent study by Auerbach et al. (20) found that diagnostic errors directly contributed to the death of 121 patients (6.6%) out of 1,863 patients in Class I, highlighting the harmful effects of clinical diagnostic errors. Both their study and ours highlight the limitations of modern diagnostic tools in determining the cause of death. Some of these diagnostic tools have issues with specificity and sensitivity, leading to high rates of false positives and false negatives and contributing to clinical errors and discrepancies. Therefore, caution should be taken in patient care to ensure quality assurance (20).

However, the discrepancy rate in the current study is consistent with studies by Winters et al. (21) and Travora et al. (22), which showed clinical-autopsy discrepancy rates to be fairly stable over several decades at 10-30%. The meta-analysis by Kaveh et al. (19) also suggested that the persistence of major discrepancies may be more systemic than merely technological inadequacies. This highlights that despite advanced imaging capabilities, laboratory methods, and clinical decision support systems, fundamental challenges in clinical reasoning and diagnostic processes remain inadequately addressed (21).

Clinicopathological factors like sex, gender, race, rare diseases, and seropositive status increase the risk of major discrepancies. The mean age at death of 39.2 years, with 82.35% of patients older than 18 years, indicates that diagnostic discrepancies predominantly affected adult patients. While Fisher's exact test showed no evidence of association between age and discrepancy class (*p* = 0.716), the relatively young mean age raises concerns about premature mortality potentially linked to diagnostic failures (19). The female predominance (68.63%) in this cohort may reflect the substantial contribution from obstetrics and gynecology (33.39% of autopsy requests), where conditions such as ruptured uterus and choriocarcinoma contributed to fatal outcomes.

The distribution of autopsy requests reveals important information on the persistent diagnostic challenges across different specialities. Internal medicine (35.29%) and obstetrics and gynecology (33.39%) made the most requests. This suggests that these departments deal with complex, multisystem pathology where diagnostic uncertainty is common.

The high number of Class I diagnostic errors in internal medicine patients likely reflects the diagnostic complexity of managing patients with multiple comorbidities and atypical or overlapping clinical features (21). In obstetrics and gynecology, advocating for autopsy on all maternal deaths may be in keeping with the Department of Health's Key National Health Priorities (2025–2030), particularly the goal of reducing maternal and child mortality (23).

Pulmonary diseases accounted for the largest proportion of undiagnosed cases (39.22%), all categorized as Class I discrepancies (*p* = 0.002), raising significant concerns about patient safety. These findings align with the literature, which indicates that pulmonary pathology is among the most commonly misdiagnosed categories at autopsy (22, 24). Winters et al. (21) identified pneumonia, pulmonary embolism, and other respiratory conditions as significant factors contributing to diagnostic errors, particularly in emergency and acute care settings.

There are several reasons for the high rate of missed pulmonary diagnoses. Respiratory symptoms are notoriously non-specific and can overlap with numerous conditions, complicating clinical differentiation. Bronchopneumonia and interstitial lung disease may exhibit subtle radiological findings that can be obscured by comorbidities or misinterpreted as chronic alterations in individuals with pre-existing lung conditions. Pulmonary embolism is still one of the most difficult diagnoses to make in a clinical setting. The majority of fatal pulmonary emboli are not suspected prior to death (22, 24).

The equal distribution of malignancies across Class I and Class II discrepancies (*n* = 10, 19.61% total) reveals a biphasic pattern of diagnostic failure in cancer detection. The Class I malignancies, adenocarcinomas of the pancreas and endometrium, choriocarcinoma, and poorly differentiated carcinomas, represent cancers where earlier detection could have enabled curative interventions or prolonged survival (21). On the other hand, Class II malignant discrepancies more probably represent advanced or aggressive tumors because even an accurate antemortem diagnosis would not have altered the fatal outcome. Recent literature emphasizes that missed malignancies continue as an important source of diagnostic error, with pancreatic adenocarcinoma and choriocarcinoma particularly prone to late diagnosis owing to their nonspecific presentations and mimickers. Plasmablastic lymphoma and metastatic basal cell carcinoma findings in autopsy diagnoses emphasize how rare or atypical presentations can escape clinical detection, even after a full workup has been done (25, 26).

All cardiovascular discrepancies (13.73%) were classified as Class I; consequently, conditions such as arrhythmogenic right ventricular cardiomyopathy, hypertrophic cardiomyopathy, and infective endocarditis are clinically unsuspected despite their potential treatability. This resonates with findings from prior studies reporting that structural cardiac diseases and endocarditis remain frequently underdiagnosed, particularly among younger patients and those who present with non-classic symptoms (24).

The same applies to the infectious disease category (9.80%), which includes bacterial meningitis, syphilis, and disseminated fungal infections (cryptococcosis and mucormycosis). Such situations reflect diagnostic difficulties in immunocompromised populations and in environments where opportunistic infections may mimic other conditions. The presence of disseminated mucormycosis and cryptococcosis suggests a possible missed diagnosis in patients with some degree of immunosuppression; such cases warrant high clinical suspicion and an aggressive diagnostic approach (10).

Overall, the high proportion of Class I discrepancies, both in the current study and in the literature, highlights a persistent gap in diagnostic accuracy and reinforces the continued value of autopsy as a quality assurance tool (19). Emphasizing the need for healthcare systems to invest resources in initiatives to improve diagnostic safety, including enhanced training in clinical reasoning, interdisciplinary case reviews, and structured protocols for high-risk diagnostic presentations (24).

Newer models indicate that diagnostic errors stem from a complex interplay among cognitive biases, system-level factors, and a lack of feedback mechanisms. Feedback from autopsies can reveal knowledge gaps, drive improvements in systems, and help organizations learn (7, 21, 27).

Declining global autopsy rates present a missed opportunity to improve quality and advance medical education (27).

The disease pattern observed in this study highlights areas where diagnostic capability should be enhanced. The high burden of misdiagnosis of pulmonary disease underscores the importance of access to advanced pulmonary diagnostics in healthcare facilities. Similarly, the significant cancer misdiagnosis rate may translate to a need for improved access to histopathological services, advanced imaging, and tumor marker panels (21, 25, 26).

For infectious diseases, particularly disseminated fungal infections among immunocompromised patients, enhanced microbiological diagnostic capabilities, including molecular diagnostics and fungal biomarker testing, are essential (10). Resource-limited settings may face specific challenges in detecting rare or complex conditions, necessitating good referral networks and consultative pathology services (24).

The persistence of diagnostic discrepancies across decades emphasizes that technological advancements alone are insufficient without parallel improvements in clinical reasoning and diagnostic thinking (19). Medical education must emphasize the generation of differential diagnoses, cognitive debiasing strategies, and systematic approaches to diagnostic uncertainty (22). Clinical-pathological multidisciplinary meetings, once a cornerstone of medical education, have declined in parallel with autopsy rates; however, they remain indispensable for teaching diagnostic reasoning and understanding disease presentation, thereby increasing the index of suspicion in unsuspecting cases (6, 8, 27).

The study demonstrated a statistically significant association between autopsy diagnosis and discrepancy class (*p* = 0.002), but this should be interpreted with caution, as no evidence of association was observed between discrepancy class and demographic variables such as age and sex suggesting that diagnostic error in this study was more strongly related to disease-related and clinical presentation factors than to patient characteristics. However, the relatively small sample size may have limited the ability to detect more subtle associations.

More robust results could be expected from multi-center studies with larger samples concerning factors predicting diagnostic discrepancies.

Lessons from diagnostic discrepancies should be included in continuing medical education programs, using real cases to illustrate common pitfalls and atypical presentations. The findings of this study support the need for improved diagnostic processes, particularly in high-risk conditions such as pulmonary, cardiovascular, and infectious diseases, and emphasize the importance of maintaining autopsy services as part of clinical governance and medical education.

## 6 Conclusion

This study highlights that major differences between clinical and autopsy diagnoses remain a significant quality issue, with Class I differences indicating potentially avoidable deaths. Pulmonary diseases were the most frequent source of diagnostic discrepancy and were significantly associated with Class I errors, while malignancies and cardiovascular and infectious diseases also contributed meaningfully to the overall burden of missed or delayed diagnoses. Within the limits of a single-center study and a relatively small sample size, these results reinforce the continued value of autopsy examination in assessing diagnostic accuracy and identifying areas of clinical uncertainty. Autopsy findings provide important insight into patterns of diagnostic error and may help guide local efforts to strengthen diagnostic processes, particularly in high-risk clinical presentations.

Overall, the study supports the continued relevance of autopsy as a quality assurance tool and underscores its role in identifying diagnostic challenges, informing improvements in clinical diagnosis, and ultimately contributing to improved patient management and outcomes.

## Data Availability

The original contributions presented in the study are included in the article/supplementary material, further inquiries can be directed to the corresponding author.
